# Defining the transcriptional and biological response to CDK4/6 inhibition in relation to ER+/HER2- breast cancer

**DOI:** 10.18632/oncotarget.11588

**Published:** 2016-08-18

**Authors:** Erik S. Knudsen, Agnieszka K. Witkiewicz

**Affiliations:** ^1^ Department of Medicine, University of Arizona, Tucson, AZ, USA; ^2^ University of Arizona Cancer Center, University of Arizona, Tucson, AZ, USA; ^3^ McDermott Center for Human Growth and Development, University of Texas Southwestern Medical Center, Dallas, TX, USA; ^4^ Department of Pathology, University of Arizona, Tucson, AZ, USA

**Keywords:** CDK4/6, breast cancer, RB-pathway, PAM50, molecular subtypes

## Abstract

ER positive (ER+) and HER2 negative (HER2-) breast cancers are routinely treated based on estrogen dependence. CDK4/6 inhibitors in combination with endocrine therapy have significantly improved the progression-free survival of patients with ER+/HER2- metastatic breast cancer. Gene expression profiling in ER+/HER2- models was used to define the basis for the efficacy of CDK4/6 inhibitors and develop a gene expression signature of CDK4/6 inhibition. CDK4/6 inhibition robustly suppressed cell cycle progression of ER+/HER2- models and complements the activity of limiting estrogen. Chronic treatment with CDK4/6 inhibitors results in the consistent suppression of genes involved in cell cycle, while eliciting the induction of a comparable number of genes involved in multiple processes. The CDK4/6 inhibitor treatment shifted ER+/HER2- models from a high risk (luminal B) to a low risk (luminal A) molecular-phenotype using established gene expression panels. Consonantly, genes repressed by CDK4/6 inhibition are strongly associated with clinical prognosis in ER+/HER2- cases. This gene repression program was conserved in an aggressive triple negative breast cancer xenograft, indicating that this is a common feature of CDK4/6 inhibition. Interestingly, the genes upregulated as a consequence of CDK4/6 inhibition were more variable, but associated with improved outcome in ER+/HER2- clinical cases, indicating dual and heretofore unknown consequence of CDK4/6 inhibition. Interestingly, CDK4/6 inhibition was also associated with the induction of a collection of genes associated with cell growth; but unlike suppression of cell cycle genes this signaling was antagonized by endocrine therapy. Consistent with the stimulation of a mitogenic pathway, cell size and metabolism were induced with CDK4/6 inhibition but ameliorated with endocrine therapy. Together, the data herein support the basis for profound interaction between CDK4/6 inhibitors and endocrine therapy by cooperating for the suppression of cell cycle progression and limiting compensatory pro-growth processes that could contribute to therapeutic failure.

## INTRODUCTION

ER+/HER2- represents the most common routine diagnosis of breast cancer representing approximately 65-70% of patients [1]. Such tumors are conventionally treated with endocrine therapy [2–5]. These regimens are overall effective; however, there remains a significant risk of recurrence for patients. Patients that exhibit highly proliferative tumors or are categorized as high risk by multi-gene tests are at particularly high-risk for recurrence and incur a clinical benefit from post-surgical chemotherapy [6–9]. However, even patients with relatively indolent tumors remain at elevated risk for recurrence through their life compared to the general population.

Unfortunately, in spite of extensive surveillance and monitoring, recurrence occurs as metastatic disease in approximately 25% of cases within 5 years [10, 11]. Typically, metastatic ER+/HER2- breast cancer is treated with subsequent lines of endocrine therapy (e.g. fulvestrant); however, disease control is typically not durable with progression occurring in most patients [12–14]. These features of the clinical disease have prompted the development of combination therapies to enhance the durability of endocrine therapy [4].

Endocrine therapy functions in part through suppression of cell cycle progression [10, 15–17]. In ER+/HER2- models, endocrine therapy results in cell cycle arrest in G1 that is accompanied by the inhibition of cyclin D1 expression and the attenuation of cyclin-dependent kinase (CDK) activity [17]. This inhibition of CDK results in the dephosphorylation of RB which represses the transcription of multiple genes required for cell cycle progression [18]. In preclinical models of resistance to endocrine therapy, this signaling axis is compromised such that cell cycle progression occurs irrespective of estrogen receptor signaling [19, 20]. These findings are germane to the clinical experience as suppression of proliferation measured by Ki67 with pre-surgical endocrine therapy is a predictive determinant for long-term therapeutic response [21].

CDK4/6 inhibitors have been developed by multiple pharmaceutical companies and are potent inhibitors of proliferation in many tumor types [22, 23]. In particular, they are highly effective at inhibiting the growth of ER+ models [19, 24–26]. Additionally, they have been shown to both cooperate with endocrine therapy and remain functional in models that are resistant to endocrine therapy [19, 24–26]. Based on these data, clinical trials were initiated that demonstrated a significant impact of CDK4/6 inhibitors on the progression of metastatic ER+/HER2- breast cancer in combination with endocrine therapy [12, 27]. These findings lead to the FDA approval of palbociclib in combination with endocrine therapy for the treatment of ER+/HER2- metastatic breast cancer. In spite of the clinical data, the impact of CDK4/6 inhibition on breast cancer models and the significance related to patient subtypes and signaling pathways remains surprisingly scant.

## RESULTS

**Figure 1:**
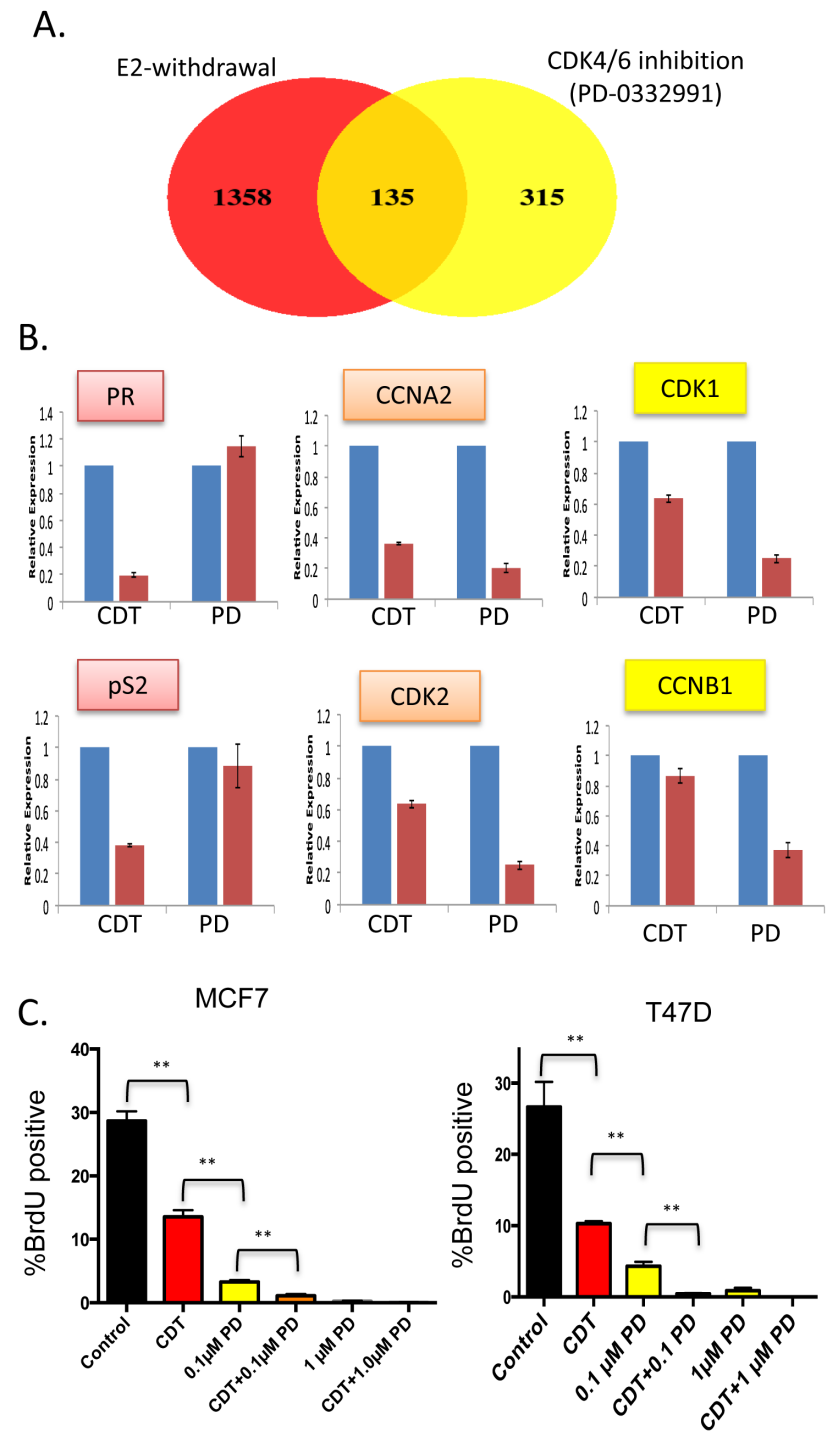
Distinct gene regulation by CDK4/6 inhibition and estrogen withdrawal—cooperation for suppression of cell cycle (**A)** Venn diagram showing the overlap in genes modified by greater than 1.5-fold and *p* < 0.05 in MCF7 cells treated with PD-0332991 *vs*. MCF7 challenged with estrogen withdrawal. **B.** Relative expression of select genes that are estrogen-specific, similarly repressed, or preferentially repressed with PD-0332991. **C.** BrdU incorporation of MCF7 and T47D cells treated with the indicated agents. CDT—charcoal dextran treated serum lacking steroid hormones, PD—the CDK4/6 inhibitor PD-0332991 (**p < 0.01).

Initially, we explored the acute impact of CDK4/6 inhibition relative to estrogen withdrawal. The ER+/Her2- cell line MCF7 was cultured in standard growth media and treated with the CDK4/6 inhibitor PD-0332991 for 24 hours. Differentially regulated genes were determined by microarray analysis and compared against the expression profile of MCF7 cells that were deprived of estrogen (Figure 1A). This model mimics endocrine therapies that impinge on endogenous estrogen physiologically (e.g. aromatase inhibitors). As expected, estrogen withdrawal had a dramatic impact on gene expression with over 1,000 genes exhibiting a greater than 1.5 fold change in level with *p* < 0.05. In contrast, PD-0332991 resulted in the altered expression of ~450 genes (Figure 1A). The cessation of estrogen signaling impacted known ER target genes such as TFF1 (pS2) and the progesterone receptor (PR) that were not affected by treatment with PD-0332991 (Figure 1B). In contrast, there were many genes that were significantly repressed by both estrogen withdrawal and PD-0332991 using the cutoffs employed. The majority of these genes were involved in cell cycle regulation (Figure 1B). Interestingly, PD-0332991 generally had a larger effect on the repression of such genes; additionally there were a number of cell cycle regulatory genes that were only marginally repressed by estrogen withdrawal relative to PD-0332991 treatment (Figure 1B). Consistent with these findings, while estrogen withdrawal suppressed cell cycle progression of MCF7 and T47D cells, the impact of 100 nM PD-0332991 was more significant (Figure 1C). This cooperation is likely relevant to the therapeutic efficacy of combinatorial treatment (Figure 1C).

**Figure 2:**
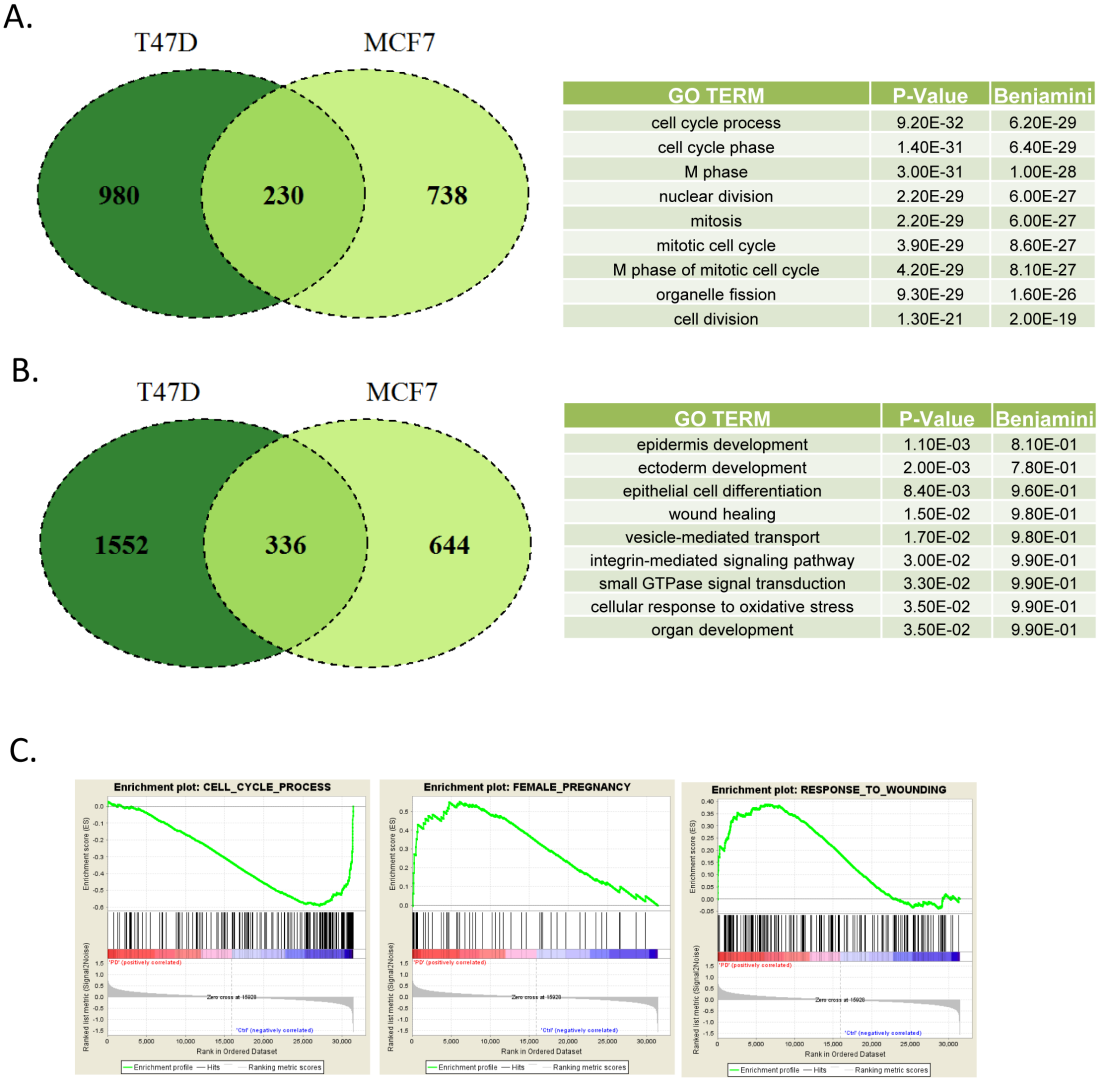
Defining CDK4/6 inhibition signature in ER+/Her2- models **A** Venn diagram showing the overlap in genes repressed by greater than 1.5-fold and *p* < 0.05 in MCF7 and T47D cells treated with PD-0332991 for 120 hours. Top gene ontologies were determined for the genes repressed in both models. **B.** Venn diagram showing the overlap in genes induced by greater than 1.5-fold and *p* < 0.05 in MCF7 and T47D cells treated with PD-0332991 for 120 hours. Top gene ontologies were determined for the genes that were induced in both the models. **C.** Gene set enrichment analysis of selected terms associated with transcriptional repression (e.g. cell cycle) *vs*. activation (e.g. pregnancy and wound healing).

Since acute effects may not necessarily be important to the clinical efficacy, and may minimize compensatory downstream signaling, T47D and MCF7 cells were treated with PD-0332991 for 120 hours. This treatment resulted in a large number of alterations in gene expression (Figure 2). In particular, there were 230 genes that were commonly repressed in both T47D and MCF7 cells (Figure 2A). This repression signature was strongly enriched for cell cycle dependent processes as determined by gene ontology, and included genes present in previously characterized RB and E2F signatures [18, 28, 29] consistent with the known action of CDK4/6 (Figure 2A). As opposed to acute treatment where there were few induced genes, a large number of genes were activated by the prolonged exposure to CDK4/6 inhibitors (Figure 2B). A total of 336 genes were upreguated in both T47D and MCF7 cells. Interestingly, this signature was not strongly associated with any specific gene ontology. Gene set enrichment analysis demonstrated enrichment for cell cycle with repression, while response to wounding and female pregnancy were enriched in gene activation (Figure 2C).

**Figure 3:**
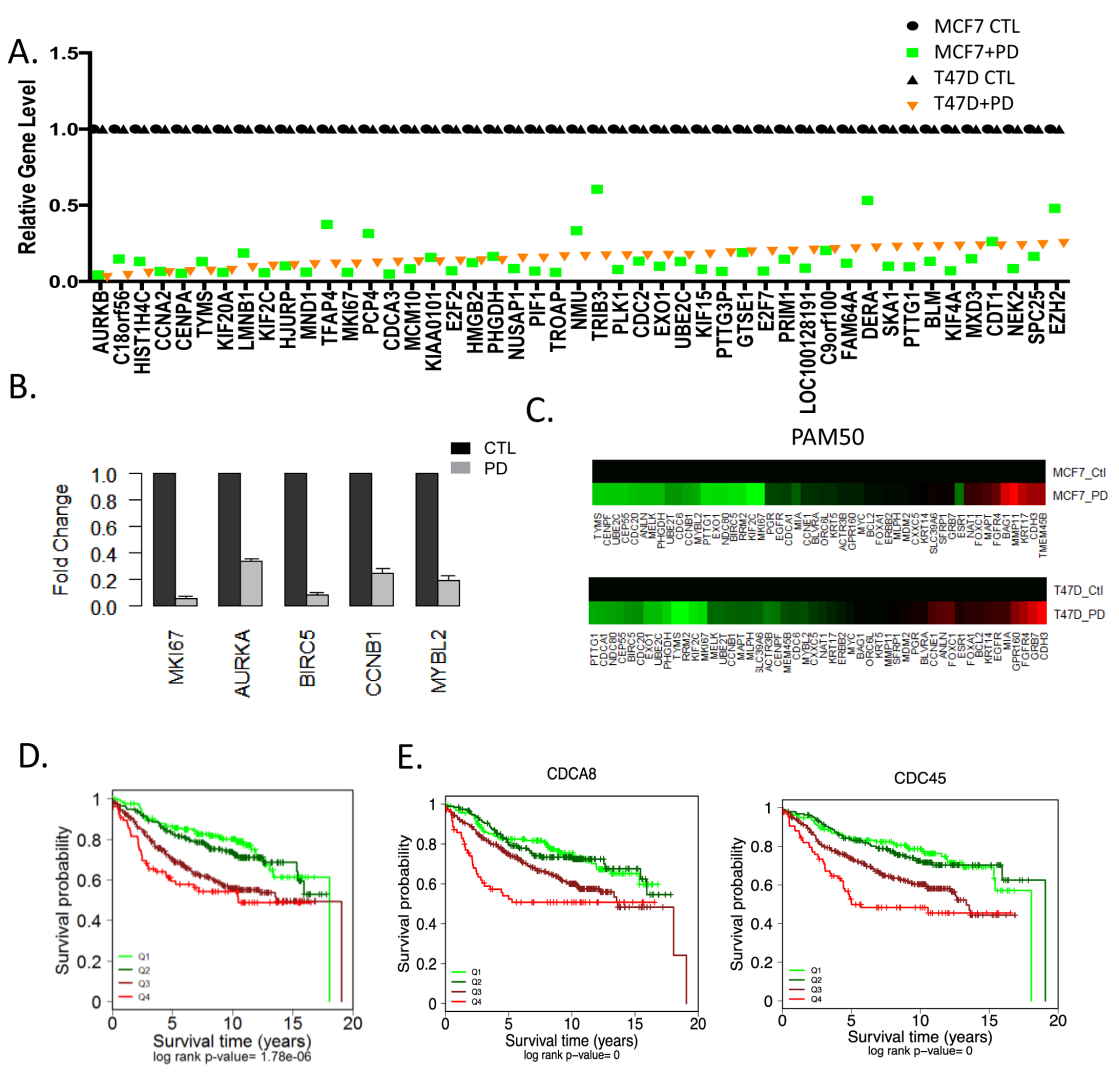
Transcriptional repression by CDK4/6 inhibition and impact on luminal subtypes **A** The levels of top repressed genes in T47D cells are presented by rank order. Expression in both T47D and MCF7 cells is shown as denoted by the legend. **B.** Relative level of genes that make up the OncotypeDX proliferation module were evaluated in MCF7 cells untreated (black bars) and treated with PD-0332991 (gray bars). **C.** Relative expression of the PAM50 genes untreated and treated with PD-0332991 in MCF7 and T47D cells. Data indicate the consistent suppression of proliferation associated genes that are associated with risk of recurrence. **D.** Kaplan-Meier analysis of the survival data from 967 ER+/Her2- tumors stratified based on the level of the 230 gene CDK4/6 repression signature. The quartiles associated with transcriptional repression are shown statistical significance was determined by log-rank of the quartiles. The highest expression (Q4) of gene expression is associated with worse prognosis **E.** Select genes that are significantly repressed by CDK4/6 inhibition in MCF7 and T47D cells and have potent prognostic activity in ER+/HER2- breast cancer are shown. The highest expression (Q4) of gene expression is associated with worse prognosis.

Analysis of transcriptional repression at the gene level illustrated a profound inhibition of the expression of multiple cell cycle regulatory genes with CDK4/6 inhibition in both T47D and MCF7 cells (Figure 3A). Visual inspection of the genes revealed that veritably all of the genes that are in the OncotypeDx proliferation module associated with recurrence are repressed by PD-0332991 (Figure 3B). These data suggest that treatment with PD-0332991 converts high-risk to low risk ER+/HER2-. Consistent with this concept, there were equivalent alterations in the gene expression within the PAM50 that would shift the behavior of T47D and MCF7 into the luminal A subtype of breast cancer (Figure 3C). The common repressed genes greater than 1.5-fold (*p* < 0.05) were used to stratify ER+/Her2- breast cancer cases, and were strongly associated with prognosis (Figure 3D). Similarly, individual repressed genes (e.g. CDC45 and CDCA8) harbored prognostic significance (Figure 3E). These data illustrate that the repression signature of CDK4/6 inhibition has potent prognostic activity, suggesting that treatment with CDK4/6 inhibition would be associated with a switch to a form of ER+ breast cancer with a generally improved prognosis.

**Figure 4:**
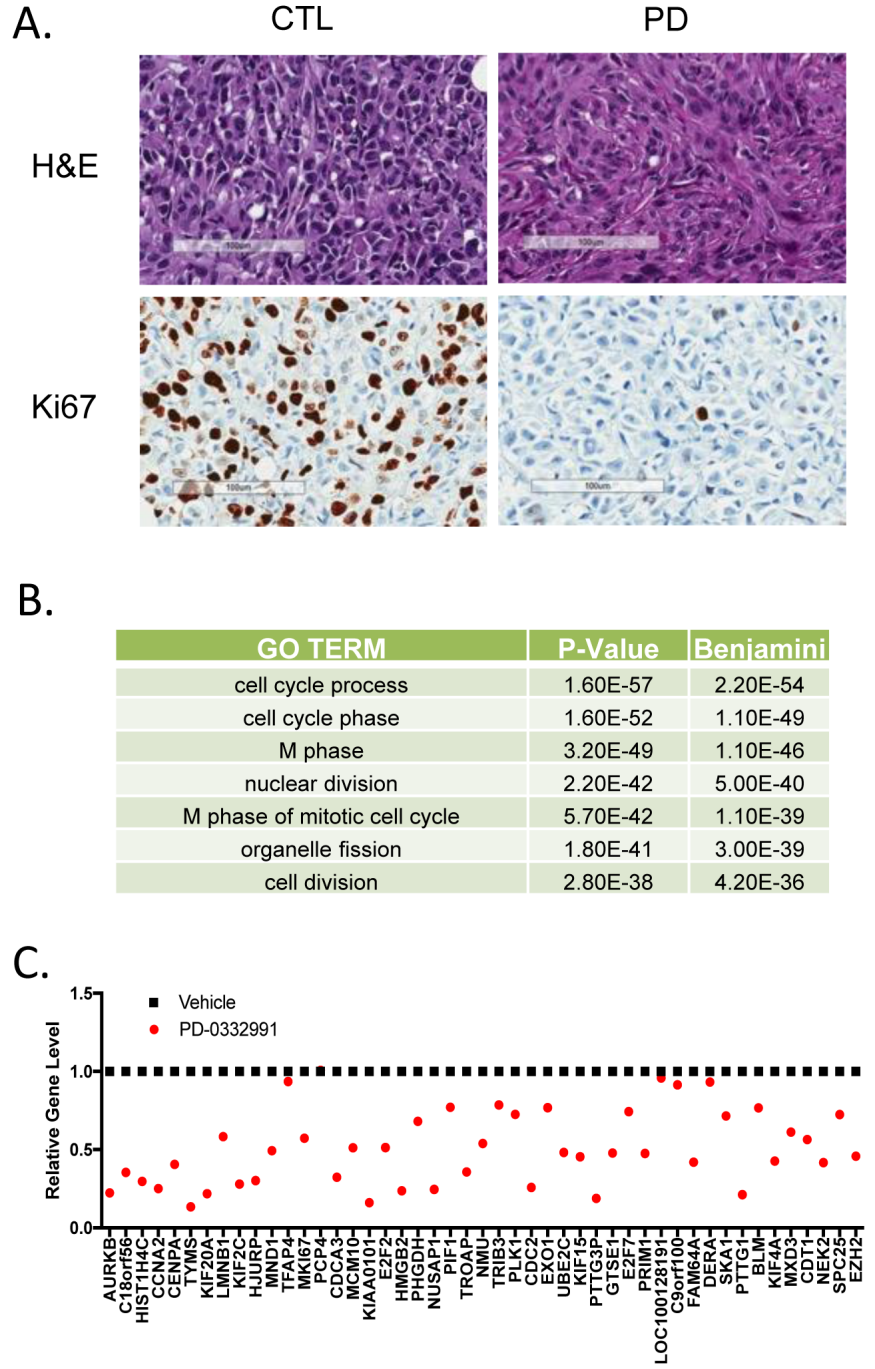
Conservation of CDK4/6 inhibitor gene repression in xenografts and different breast cancer subtypes **A** Histological analysis of MDA-MB-231 orthotopic xenograft controls or treated with PD-0332991 orally for seven days. Representative hematoxylin/eosin stained sections and Ki67 staining are shown. Scale bar is 100 μm. **B.** Gene ontology was performed on 278 genes that were repressed 1.5-fold with a *p*-value < 0.05. **C.** Specific analysis of the expression levels of top repressed genes in cell culture models.

To determine the generality and physiological significance of these repressed genes, we employed an aggressive orthotopic xenograft model. Notably, MDA-MDA-MB-231 cells (derived from triple negative breast cancer) were introduced into the mammary fatpad of NOD/SCID mice. When tumors were palpable (~300 mm^3^) mice were randomized to treatment with palbociclib (100 mg/kg) daily by oral gavage. After 7 days of treatment, mice were sacrificed and tumor tissue was assessed. As shown in (Figure 4A), there was a pronounced inhibition of Ki67 by treatment with PD-0332991 *in vivo*. RNA was extracted from the tumors from three independent mice and subjected to microarray analysis. The repressed genes fell into gene ontologies that were veritably identical to the gene expression programs observed from the ER+/HER2- models grown in culture (Figure 4B). At the gene level the overlap in repressed genes was highly significant (*p <*1x10 ^−10^), consonantly when top repressed genes in T47D cells were evaluated the majority of them were repressed greater than 1.5-fold (Figure 4C). Interestingly, genes that were not repressed in the xenografts (e.g. TFAP4 and DERA) were weakly repressed in MCF7 cells and have roles outside of the cell cycle. Together, these data indicate that there is a highly conserved gene expression program repressed by CDK4/6 inhibitors that transcends breast cancer subtypes.

**Figure 5:**
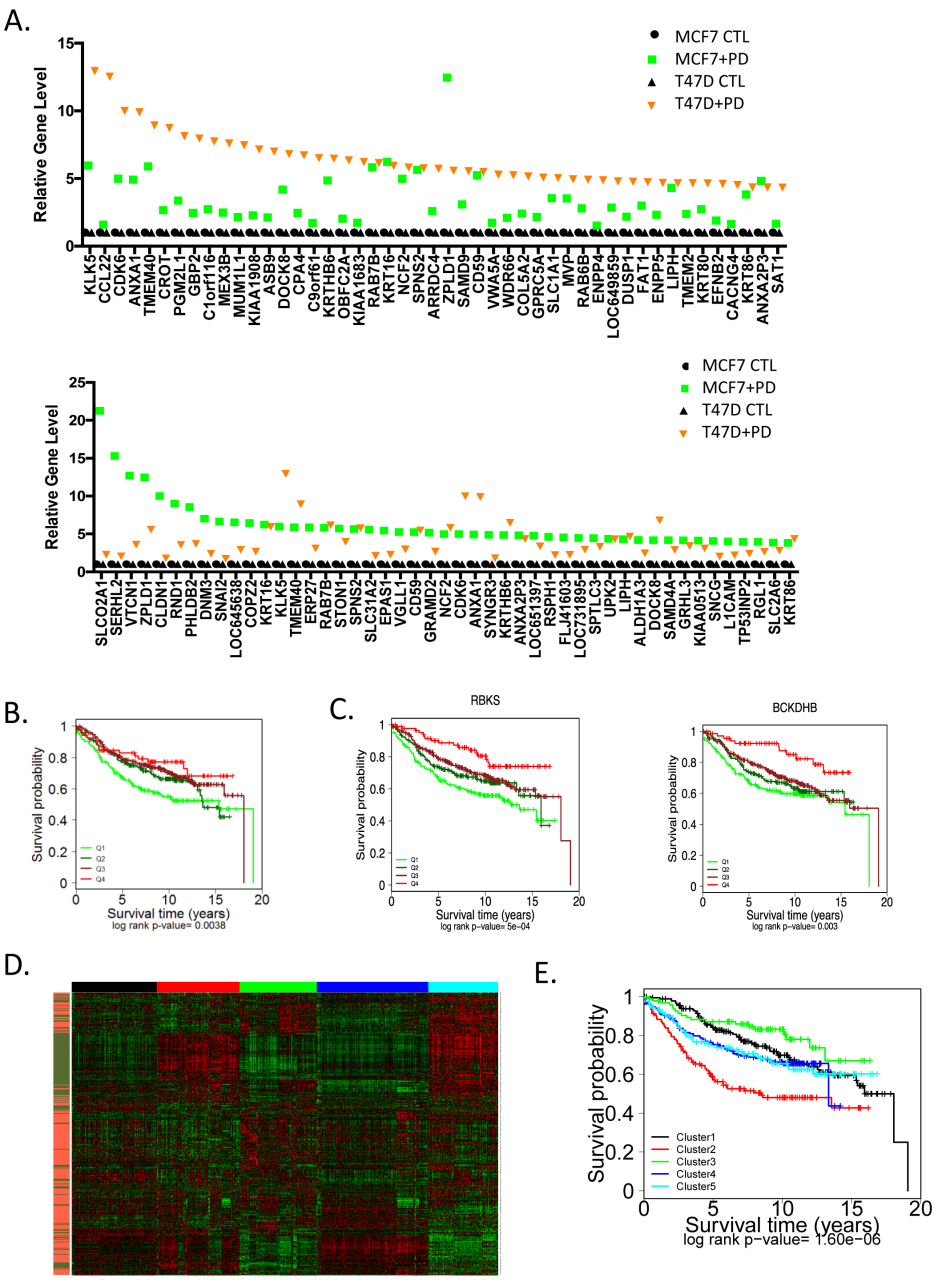
Unexpected impact of genes upregulated through CDK4/6 inhibition on prognosis **A** The levels of top induced genes in MCF7 or T47D cells are presented by rank order. **B.** The common CDK4/6 inhibitor induced signature (336 genes) was used to stratify the expression data from 967 ER+/HER2- cases. Survival outcomes were determined by Kaplan-Meier analysis **C.** Select genes from the signature that are associated with prognosis are presented. Statistical analysis was determined by log-rank analysis. **D.** Heatmap of K-Means clustering including upregulated (orange) and downregulated (green) genes from the CDK4/6 inhibitor signature, the 5 clusters are denoted by color bar at the top of the heatmap. **E.** The indicated K-means clusters were used to determined the association with prognosis by Kaplan-Meier analysis. Statistical signifiace was determined by log-rank analysis.

The genes induced by PD-0332991 were considerably more variant in function and in gene expression level between T47D and MCF7 (Figure 5A). In spite of this heterogeneity, the common gene signature induced by PD-0332991 was also associated with prognosis in ER+/HER2- breast cancer (Figure 5B). In particular, elevated levels of this signature are associated with improved outcome and this effect was again recapitulated by single genes within the signature (Figure 5C). These data suggest that with inhibition of CDK4/6 there is both the suppression of genes associated with cell cycle that denote recurrence, and the induction of a class of genes that are associated with improved survival.

To interrogate the composite induced and repressed signatures, they were employed in K-Means clustering to define subtypes of ER+/HER2- breast cancer (Figure 5D). Five clusters were generated, wherein clusters 2 (red) and 5 (teal) were dominated by proliferative genes and would be generally categorized as luminal B. The inclusion of the genes upregulated by CDK4/6 inhibition generated a prognostic discrimination between these groups (Figure 5E). These data suggest that the dual effects of CDK4/6 inhibition impact biological features of ER+/HER2- breast cancer and can elicit functional effects beyond suppression of cell cycle progression. In contrast, the luminal A clusters 1 (black), 3 (green), and 4 (blue) maintained relative good prognosis, although again the additional information associated with upregulated genes lead to discrimination also within this group (e.g. cluster 3 *vs*. cluster 4).

**Figure 6:**
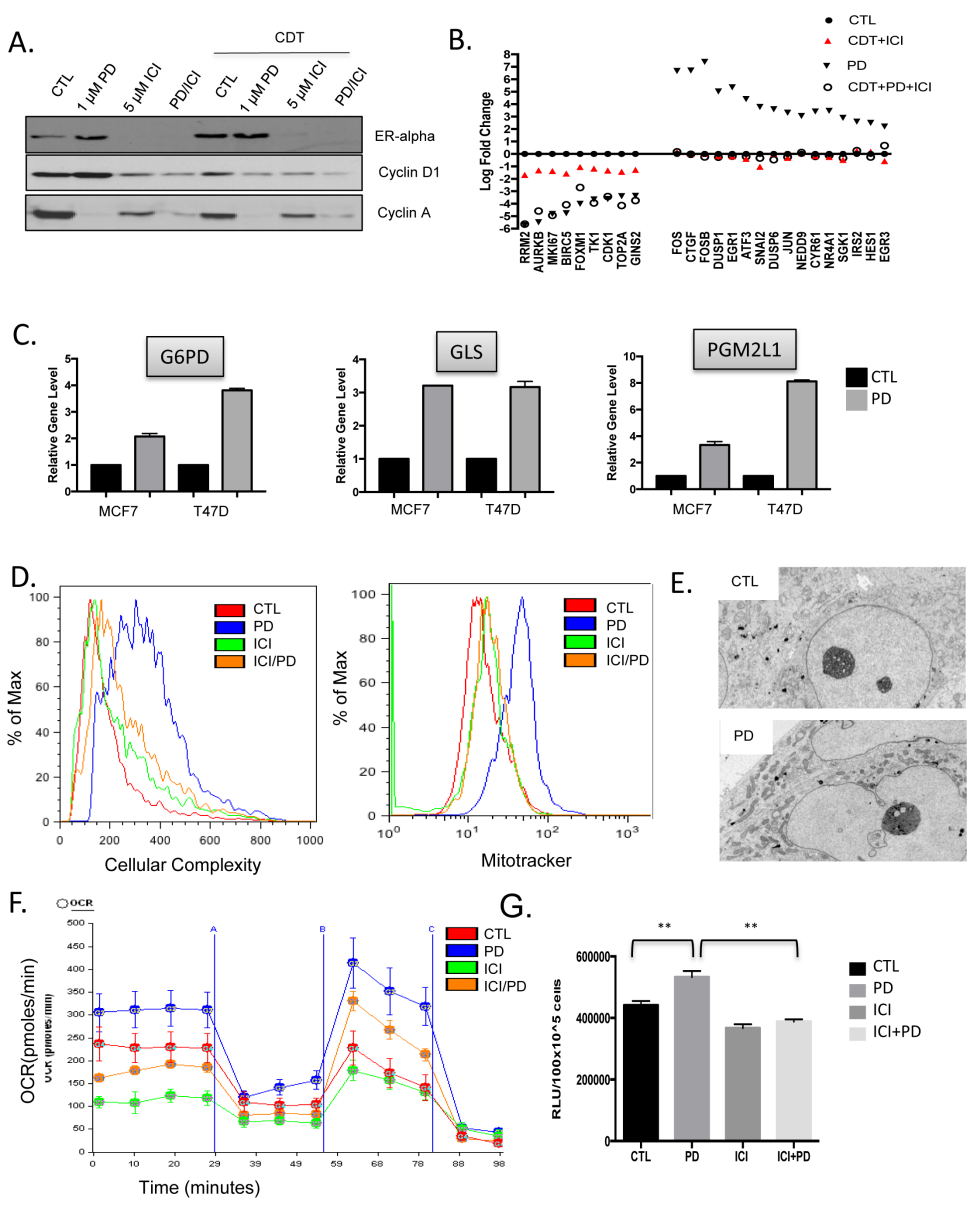
CDK4/6 induction of cellular growth and metabolism are ameliorated by endocrine therapy **A** The levels of the estrogen receptor (ER), cyclin D1, and cyclin A were determined by western blotting under the indicated conditions. **B.** The expression of the indicated genes was determined by RNA sequencing analysis from T47D cells treated with the indicated therapeutics (CDT—charcoal dextran treated serum, ICI—fulvestrant, or PD—the CDK4/6 inhibitor PD-0332991. The relative gene expression is shown in reference to standard growth conditions. **C.** The impact of CDK4/6 inhibition on the expression of select metabolic genes is shown for MCF7 and T47D cells. **D.** Impact of the indicated therapeutics on cellular complexity was determined by flow cytometry and impact on mitochondria was determined by mitotracker staining and flow cytometry. **E.** Representative transmission electron micrographs showing the accumulation of mitochondria in cells treated with PD-0332991. **G.** Impact of the indicated treatments on oxidative metabolism as determined by oxygen consumption rate (OCR) analysis. **H.** Relative ATP present in T47D cells following the indicated treatment (**p < 0.01).

One of the known features of the response to CDK4/6 inhibitors is the maintenance or accumulation of cyclin D1 expression [30, 31]. This phenomenon was clearly observed in T47D cells; however, this effect on cyclin D1 was reversed by the treatment with endocrine therapies (Figure 6A). To understand the basis for this response, RNA sequencing was performed following short term treatment with PD-0332991 *vs*. fulvestrant and estrogen withdrawal (ICI+CDT) *vs*. combination treatment (PD+ICI+CDT) in T47D cells (Figure 6B). Under these conditions it was clear that combined endocrine therapy represses specific cell cycle dependent genes, albeit PD-0332991 was more potent at mediating the transcriptional repression (Figure 6B). Surprisingly a large number of conventional genes that are downstream from mitogenic signaling (e.g. FOS, EGR1, JUN, DUSP6) were induced as a consequence of PD-0332991 treatment (Figure 6B). This response was not just a feature of cell cycle inhibition, as endocrine therapy did not induce these genes. Furthermore, the effect of endocrine therapy was dominant to PD-0332991 and blocked the induction of such genes. These combined data suggest that CDK4/6 inhibition elicits a compensatory mitogenic response in ER+/HER2- models.

Mitogenic signals serve to both drive cell cycle progression and fuel cell growth/metabolism for subsequent cell division [32]. In fact, key genes involved in glucose (G6PD and PGM2L1) and glutamine (GLS) metabolism were induced (Figure 6C). Analysis of cellular complexity, which is an indirect measure of cell size and organelles in the cell, demonstrated an increase with PD-032991 treament that was antagonized by treatment with ICI (Figure 6D). Similarly by mitotracker straining and electron microscopy, the number of mitochondria increased with PD-0332991 treatment in T47D cells (Figure 6D) and 6E). However, treatment with endocrine therapy blunted this effect similar to the observations on gene expression (Figure 6D) and 6E). Correspondingly, T47D cells treated with PD-0332991 exhibit higher activity for oxygen consumption rate and ATP levels relative to control or fulvestrant treated cells (Figure 6E) and 6F). Thus, while CDK4/6 inhibition resulted in stimulated metabolic acitivty, ER antogonists were dominant and inhibited this response.

## DISCUSSION

Together, the data herein provide insight into the impact of CDK4/6 inhibition on ER+/HER2- models of breast cancer and the relevance to breast cancer clinical outcomes.

The canonical function of CDK4/6 inhibition is to elicit the functional activation of the RB tumor suppressor and block subsequent cell cycle progression [22, 23]. As shown here, and consistent with the work of others, CDK4/6 inhibitors are very potent at inhibiting the proliferation of ER+/HER2- models [24]. In general, the depth of cell cycle inhibition exceeds that of endocrine therapy in the models used herein. However, at lower doses of CDK4/6 inhibitor there is clear cooperation with endocrine therapy for this endpoint and suggests a simple basis for the success of the combination that has been observed clinically. One of the interesting features of CDK4/6 inhibitors is the accumulation/maintenance of cyclin D1 expression. This is distinct from most other targeted agents, as one of the key points of cooperation between CDK4/6 inhibition and endocrine therapy is likely at this level in the interface of mitogenic signaling controlling the accumulation of cyclin D1 and subsequent cell cycle progression coordinated by CDK4 and CDK6 activity [30, 31].

Gene expression profiling with targeted panels have transformed the clinical care of ER+/HER2- breast cancer [33–35]. High-risk for recurrence of disease is denoted by the luminal B subtype which is largely a reflection of the proliferative status of the tumor [6–8, 35]. CDK4/6 inhibition yields the repression of genes that are associated with risk of recurrence in ER+/HER2- breast cancer and functionally converts a luminal B like pattern of gene expression towards a luminal A form of disease. This could be similarly important in considering both the interaction with endocrine therapy, and also considerations relevant to chemotherapy as luminal A breast cancer exhibits little benefit from chemotherapy [36]. While downregulated genes are easy to ascribe to the conventional function of CDK4/6 in phosphorylating RB [37, 38], the upregulated genes largely defy a simple explanation. Surprisingly, not only were the repressed genes associated with prognosis, but so too were the genes that were upregulated by CDK4/6 inhibition. This finding suggests that there is an indirect feature of CDK4/6 inhibition that leads to improved prognosis in breast cancer. The mechanisms through which such upreguated genes could contribute to outcome is currently unknown. However, they could reflect a different, previously unappreciated, subset of breast cancer. Importantly, by combining both elements of CDK4/6 response, it is possible to parse the current proliferative/non-proliferative subtypes into additional prognostic groups that could have significance in assessing risk of recurrence in ER+/HER2- breast cancer.

One of the surprising features of treatment with CDK4/6 inhibitors was the induction of genes that are conventionally associated with cell growth/proliferation. Emerging data from several models suggest that CDK4/6 inhibitors yield aberrant mitogenic signaling pathway activation that could contribute to resistance [31, 39]. Here we show that this mitogenic signal is conditioned by the presence of endocrine therapy, such that in the presence of estrogen blockade and fulvestrant this response is muted. Importantly, such treatment limits the expression of cyclin D1 and the accumulation of mitochondria and metabolic activity. Since tumor cell growth fuels subsequent rounds of proliferation these findings are germane to the treatment of ER+/HER2- breast cancer. The data indicate that endocrine therapy both cooperates with CDK4/6 inhibition in cell cycle control and also prevents this compensatory growth that could contribute to resistance to CDK4/6 inhibition. Futhermore, such accumulated signaling could yield rapid tumor growth with the cessation of CDK4/6 inhibitory treatment. Notably, several CDK4/6 inhibitors are given on a discontinuous schedule; therefore limiting energetics/mitogenic signaling and enforcing cell cycle inhibition through another mechanism may be particularly important. Specific studies to address this point have been limited, but could contribute to new dosing schedules to increase efficacy [40]. Together, these data underscore the importance of combination therapies with CDK4/6 inhibitors that both block cell cycle and mitogenic signaling that is becoming a generalized theme in the clinic.

## MATERIALS AND METHODS

### Cell culture and drug treatment

MCF7 and T47D cells were obtained from the ATCC. Cells were cultured routinely in DME with 10% fetal bovine serum. For drug treatments, cells were treated with PD-0332991 (Selleck Biochem) at the indicated doses (100 nM-1 μM). Treatments ranged from 24-120 hours. To mimic estrogen withdrawal, cells were washed with PBS and cultured in DME supplemented with 10% charcoal dextran treated (CDT) Serum. The ICI was delivered at the concentration of 1 μM consistent with prior studies.

### Xenograft studies

All mouse care, treatment, and sacrifice was approved by the UT Southwestern Institutional Animal Care and Use Committee (IACUC) in accordance with the National Institutes of Health (NIH) *Guide for the Care and Use of Laboratory Animals*. Female NOD/SCID mice at 6-8 weeks of age were surgically manipulated under anesthesia to reveal the mammary gland. Each mouse was injected with 1x10^6^ MDA-MB-231 cells in the mammary gland. Mice were monitored for tumor formation until palpable tumors were detected (~300 mm^3). Mice were randomized based on tumor size to control arm or treatment with PD-0332991 (palbociclib, 125mg/kg) for seven days. One day after the final administration, mice were sacrificed and tumor tissue was collected for analysis. Isolated tumor samples were fixed in 10% neutral buffered formalin for 48-72 hours, processed, and paraffin embedded. Specimens were cut to a thickness of 4mm and stained with hematoxylin and eosin or Ki67 using standard approaches.

### Microarray analysis

MCF7 AND T47D cells were treated with DMSO or PD-0332991 (1 μM) for 24 or 120 hours and subjected to microarray analysis using Illumina HumanHT-12 V4.0 expression beadchip array or Affymetrix human genome U133 chip. For the xenografts, tumor tissue from three mice were isolated and RNA was hybridized to the Illumina HumanHT-12 V4.0 expression beadchip array. The data was normalized using the robust multi-array average method (RMA) implemented in the limma Bioconductor package in R. For genes with multiple probe sets, the median expression level was used. A two tailed *t*-test was calculated to identify differentially expressed genes. Genes with absolute fold change greater than 1.5 and *p*-value < 0.05 were identified as differentially expressed. Gene ontology enrichment for PD-0332991 treated cells were obtained using gene set enrichment analysis (GSEA)using default parameters. Biological process database (c5.bp.v.5.1) from the molecular signature database (MSigDB) was used as the gene set of interest. The PAM50 genes and oncotypeDx proliferation genes were obtained from the relevant primary publications. Gene expression data GSE11324 from Carroll et al. was downloaded from gene expression omnibus and RMA normalized [41]. Differentially expressed genes for estrogen-deprived MCF7 cells (2-fold change, *p* < 0.01) at the 12 hour point were obtained using the lmFit function of limma package in R.

### Analysis of clinical breast cancer gene expression

Gene expression datasets of 2254 primary breast tumors on Affymetrix Human Genome U133 along with their clinical pathological features were collected and summarized as previously described [29]. The datasets were quantile normalized. A total of 967 ER+/HER2- cases were used for further analysis. Survival analysis was performed using the survival package in R. Expression cut points were defined based on quantiles and log rank *p*-value was obtained by fitting a cox proportional hazards model. All heatmaps were obtained using the heatmap function in R. K-means clustering on gene expression data was performed using K-means function in R using 5 clusters.

### RNAseq analysis

T47D cells were cultured in standard growth media, charcoal dextran treated serum and fulvestrant (CDT and ICI), PD-0332991, or CDT and ICI with PD-0332991 for 48 hours. RNA was isolated and subjected to paired-end 50 bp RNA sequencing. The libraries were demultiplexed and Fastq files were obtained. Tophat2 was used to align the sequences to human reference genome (UCSC hg19) and BAM files were obtained. Cufflinks was used to assemble the transcripts and relative expression levels were determined using cuffdiff compared to DMSO control.

### Metabolic and cell cycle analysis

Cell cycle arrest was determined using flow cytometry as we have previously published [39]. The analysis of cellular complexity, mitotracker signal, and visualization of mitochandria by transmission electron microscopy was determined as we have published [39]. The quantitation of ATP/cell was determined by cell-titerglow normalized to total protein.
